# Family functioning in families with 11-year-old children at familial high risk of schizophrenia or bipolar disorder and population-based controls: The Danish High Risk and Resilience Study VIA 11

**DOI:** 10.1017/S0033291725000200

**Published:** 2025-02-25

**Authors:** Nicoline Hemager, Ida Christine Tholstrup Gjøde, Maja Gregersen, Julie Marie Brandt, Anne Søndergaard, Mette Falkenberg Krantz, Lotte Veddum, Christina Bruun Knudsen, Anna Krogh Andreassen, Geneviève Piché, Merete Nordentoft, Aja Neergaard Greve, Anne Amalie Elgaard Thorup

**Affiliations:** 1 Copenhagen Research Centre for Mental Health, Mental Health Centre Copenhagen, Copenhagen University Hospital, Mental Health Services, Capital Region of Denmark, Copenhagen, Denmark; 2 Child and Adolescent Mental Health Center, Copenhagen University Hospital, Mental Health Services, Capital Region of Denmark, Copenhagen, Denmark; 3 The Lundbeck Foundation Initiative for Integrative Psychiatric Research, Aarhus, Denmark; 4 Department of Psychology, Faculty of Social Sciences, University of Copenhagen, Copenhagen, Denmark; 5 Psychosis Research Unit, Aarhus University Hospital, Aarhus, Denmark; 6 Department of Clinical Medicine, Faculty of Health and Medical Sciences, Aarhus University, Aarhus, Denmark; 7 Département de Psychoéducation et de Psychologie, Université du Québec en Outaouais, Saint-Jérôme, Québec, Canada; 8 Centre de Recherche Universitaire sur les Jeunes et les Familles, Québec, Canada; 9 Department of Clinical Medicine, Faculty of Health and Medical Sciences, University of Copenhagen, Copenhagen, Denmark

**Keywords:** family functioning, familial high-risk of schizophrenia, familial high-risk of bipolar disorder, population-based cohort study, psychopathology, global functioning, social functioning, McMaster Family Assessment Device, family dysfunction, risk indicators, severe mental disorders, primary caregivers

## Abstract

**Background:**

Poorer family functioning during childhood is associated with severe mental disorders in adulthood in the general population. However, family functioning is understudied in families with parental schizophrenia or bipolar disorder. We aimed to investigate family functioning in families with 11-year-old children of parents with schizophrenia or bipolar disorder compared with controls. Second, we aimed to examine associations between family functioning and levels of child psychopathology, child global functioning, and parental social functioning.

**Methods:**

In this prospective, population-based cohort study, we included 160 families with parental schizophrenia, 95 families with parental bipolar disorder, and 177 control families. Family functioning was measured with the 12-item version of the McMaster Family Assessment Device – General Functional Scale.

**Results:**

Families with parental schizophrenia (Cohen’s *d* = 0.29; *p* = .002) and parental bipolar disorder (Cohen’s *d* = 0.34; *p* = .004) had significantly poorer family functioning and a significantly higher prevalence of clinically significant family dysfunction (Cohen’s *d* range = 0.29–0.34; *p* values = .007) than control families. Across study groups, poorer family functioning was associated with higher levels of child psychopathology and poorer social functioning of the primary caregiver (*p* values < .001).

**Conclusions:**

Children in families with parental schizophrenia or bipolar disorder are at increased risk of experiencing family dysfunction, and poorer family functioning confers risk for more symptoms of child psychopathology and poorer parental social functioning. Future studies should investigate the potentially predictive value of family dysfunction in relation to later illness onset and other adverse outcomes in these populations.

## Introduction

Family functioning is closely related to and therefore important for the social, psychological, and physical development and health of individual family members (Epstein, 1978). In a clinical setting, poor family functioning can be characterized by difficulties in six dimensions of family relationships as defined by the McMaster Model of Family Functioning (Epstein, 1978; Staccini, Tomba, Grandi, & Keitner, 2015). (1) *Problem-solving* pertains to the family’s capability to resolve problems and support constructive family functioning. (2) *Communication* reflects the family’s ability to communicate in an intelligible and direct manner. (3) *Roles* refer to whether the family has formed role relationships and practices to address family tasks. (4) *Affective responsiveness* is the ability of family members to respond emotionally appropriately in various situations. (5) *Affective involvement* reflects the degree to which family members engage and show interest in the activities of other family members. (6) *Behavior control* refers to how a family exhibits and upholds behavior standards within the family (Miller, Epstein, Bishop, Keitner, 1985). Families of individuals with severe mental disorders are at increased risk of having poorer family functioning compared with families of individuals with no mental disorders (Friedmann et al., 1997) and in families of individuals with major depressive disorder, better family functioning is associated with higher recovery rates and faster recovery as well as higher levels of overall adjustment (Staccini et al., 2015). An adverse family climate (i.e., high degree of family conflict and poorer problem-solving) was associated with poor childhood mental health in a representative, cross-sectional cohort study (Wille, Bettge, Ravens-Sieberer, & BELLA Study Group, 2008) and a clinical cross-sectional study (Oltean, 2020). Finally, a disadvantageous emotional family climate in childhood was associated with the onset of affective disorders in adulthood in a prospective general population cohort study (Saarinen et al., 2023).

In a cross-sectional study of psychiatric risk, help-seeking youth with early signs of mood and anxiety or subthreshold psychotic symptoms reported poorer functioning in one aspect of family functioning (family satisfaction) compared with controls (Santesteban-Echarri et al., 2018). Moreover, family functioning has been suggested to be important for the development of emotion regulation as well as internalizing and externalizing behavior problems (Kopp, 1982; Santesteban-Echarri et al., 2018). A previous study of the current cohort suggested that poorer emotion regulation was cross-sectionally associated with higher levels of ADHD symptoms in children at familial high risk of schizophrenia and with lower levels of global functioning in children at familial high risk of bipolar disorder at 7 years of age (Spang et al., 2022). Finally, poorer family functioning was associated with poorer psychosocial functioning in a clinical sample of patients with schizophrenia (Staccini et al., 2015).

Due to complex gene–environment interactions, children born to parents with schizophrenia or bipolar disorder are at increased risk of developing the same or any mental disorder (Uher et al., 2023). Identifying modifiable risk factors such as family functioning is a prerequisite for the development of preventive strategies in the search to improve the mental health, functioning, and quality of life in children at familial high risk of these severe mental disorders (Duffy et al., 2023). However, studies on family functioning in families with parental severe mental disorders are sparse and often include small sample sizes, a broad spectrum of mental illness, and no control group (Sell, Barkmann, et al., 2021; Sell, Daubmann, et al., 2021). In a cross-sectional study of parents with any mental illness, family dysfunction rated by the ill parent, co-parent, and their children, was increased compared to a normative sample. These family member ratings were moderately associated with clinician ratings (Sell, Daubmann, et al., 2021). A longitudinal study of family functioning in families with parental bipolar disorder reported lower cohesion and adaptability and higher levels of conflict when compared with controls but not when compared with families with non-bipolar disorder parental psychopathology (Shalev et al., 2019). Moreover, the effect of parental bipolar disorder on family functioning was mediated by parental psycho-social functioning and child psychopathology. Finally, cross-sectional evidence suggests that a higher degree of family dysfunction is associated with higher levels of child mental health problems in families with any parental mental disorder (Sell, Barkmann, et al., 2021; Wiegand-Grefe, Sell, Filter, & Plass-Christl, 2019). Importantly, the relationship between family functioning and child mental health in families with parental schizophrenia or bipolar disorder may be bidirectional and in a cross-sectional design, causality cannot be inferred (Berg-Nielsen, Vikan, & Dahl, 2002).

In the current study, we aimed to characterize family functioning reported by the primary caregiver in families of 11-year-old children at familial high risk of schizophrenia (FHR-SZ) or bipolar disorder (FHR-BP) and population-based controls. Further, we aimed to investigate whether family functioning is cross-sectionally and differentially associated with dimensional psychopathology and global functioning of the child, as well as social functioning of the primary caregiver, across the three study groups.

## Method

### Participants

The current study is part of the longitudinal cohort study, the Danish High Risk and Resilience Study. At baseline, data collection took place from January 2013 to January 2016. The original cohort included 522 7-year-old children of whom 202 had one or two parents with schizophrenia spectrum psychosis (ICD-10 codes F20, F22, F25 or ICD-8 codes 295, 297, 298.29, 298.39, 298.89, 298.99), 120 had one or two parents with bipolar disorder (ICD-10 codes F30, F31 or ICD-8 codes 296.19, 296.39), and 200 were population-based controls whose parents had not been diagnosed with any of these two disorders (Supplementary Figure S1). The latter group was age-, sex-, and municipality-matched to the group of children at FHR-SZ. The group of children at FHR-BP was unmatched but did not differ in age or sex from the other two groups. Nine children (eight children at FHR-SZ and one child at FHR-BP) had two parents diagnosed with either schizophrenia or bipolar disorder. In cases where one parent had schizophrenia and the other bipolar disorder, the child was assigned to the FHR-SZ group as per the ICD-10 hierarchy. The baseline study, the VIA 7 Study, is further detailed elsewhere (Thorup et al., 2015). The current study includes data from the first follow-up study at age 11, the VIA 11 Study, which took place from March 2017 to June 2020 and included 179 children at FHR-SZ, 105 children at FHR-BP, and 181 controls (overall retention rate = 89.1%) and their parents. The participants received oral and written information about the study prior to enrollment. We obtained written consent from all participating adults as well as from the legal guardians of the participating children. All children had Danish as their first language. Further details of the VIA 11 study design are described elsewhere (Thorup et al., 2018). The parents with a diagnosis of bipolar disorder or a schizophrenia spectrum disorder in the Danish registers were defined as index parents. Finally, the parent or another legal guardian, who knew the child best or spent the most time with the child, was defined as the primary caregiver.

### Procedures

The Danish Ministry of Health granted permission to retrieve eligible participants from the Danish registers, i.e., the Danish Civil Registration System (Pedersen et al., 2006) and the Danish Psychiatric Central Research Register (Mors et al., 2011). The study was approved by the Danish Data Protection Agency and the National Committee for Health Research Ethics (NO. H-16043682). The majority of assessments were carried out at the two research sites at Copenhagen Research Centre for Mental Health, Copenhagen, and the Psychosis Research Unit, Aarhus University Hospital, Aarhus both in Denmark. In some cases, assessments were conducted in suitable surroundings in the homes of the participating families.

### Measures

#### Clinical assessments

To assess the social functioning of the primary caregiver within the past month, the assessors conducted a semi-structured interview using the Personal and Social Performance Scale (PSP) with higher scores indicating better social functioning (Morosini et al., 2000). The PSP score ranges from 1 to 100. All PSP scores were confirmed at consensus conferences with an experienced clinician and specialist in child and adolescent psychiatry (the last author) or a psychologist with a specialization in psychiatry. The child’s level of global functioning over the past month was rated by the assessors as part of a diagnostic interview and measured with the Children’s Global Assessment Scale (CGAS) (Shaffer et al., 1983) with higher scores implying higher levels of functioning. The CGAS score ranges from a minimum score of 0 to a maximum score of 100. All CGAS scores were also confirmed at consensus conferences with an experienced clinician and specialist in child and adolescent psychiatry (the last author). Finally, the child’s level of psychopathology (problem behavior) within the past 6 months was ascertained by the primary caregiver using the Child Behavior Checklist School-Age Version (CBCL) (Achenbach & Rescorla, 2001) with higher scores denoting higher levels of psychopathology ranging from a minimum score of 0 to a maximum score of 226.

#### Assessment of family functioning

We used the McMaster Family Assessment Device (FAD) – General Functional Scale (GFS) (Epstein, Baldwin, & Bishop, 1983) rated by the primary caregiver to ascertain global family functioning. The FAD has proven to be an appropriate measure of family functioning in clinical as well as research settings (Staccini et al., 2015), and this brief 12-item version of the FAD questionnaire has demonstrated good reliability and validity (Byles, Byrne, Boyle, & Offord, 1988; Miller et al., 1985). The type of questions asked are for example: ‘In times of crisis we can turn to each other’ (positive item) and ‘We avoid discussing our fears and concerns’ (negative item). The 12 items are rated on a four-point Likert scale with the response options ‘strongly agree’, ‘agree’, ‘disagree’, or ‘strongly disagree’. After reversing the six negative items, the item scores are summed and then divided by the number of items to obtain a total score ranging from 1 to 4, where higher scores denote poorer family functioning. Cases with missing items >40% were excluded. If ≤40% items were missing, the total score was calculated based on the remaining items ad modum Ryan et al. (2005). Clinical cut-off scores discriminating between healthy (total score < 2) and unhealthy family functioning (total score ≥ 2) have been developed and show adequate sensitivity and specificity (Miller et al., 1985). The cut-off scores were derived from both a theoretical perspective, i.e., a mean ≥ 2 indicates a higher proportion of family function dimensions in an unhealthy direction, and an empirical perspective based on clinicians’ interview ratings compared with the proportion of abnormal results identified by the FAD (Miller et al., 1985).

### Statistical analyses

We used one-way analysis of variance (ANOVA) for continuous data and Pearson’s chi-squared test of independence for the categorial data in the between-group comparisons of demographic and clinical characteristics and family functioning. To control for potential effects of child age, child sex, and primary caregiver’s level of education in the between-group comparisons of family functioning, we used one-way analysis of co-variance (ANCOVA) and logistic regression for continuous and categorical data respectively. To avoid a lack of independence between observations within sibling pairs, we systematically excluded the youngest sibling in each of the 12 sibling pairs (FHR-SZ, *N* = 7; FHR-BP, *N* = 4, controls = 1), who participated in this study. To investigate how family functioning (FAD-GFS) was associated with levels of social functioning of the primary caregiver (PSP) as well as global functioning (CGAS) and dimensional psychopathology/problem behavior (CBCL) of the child, we used a general linear regression model with the FAD-GFS as outcome and PSP, CBCL, CGAS, and group status as predictors. To investigate the potential effects of child age, child sex, and primary caregiver’s level of education, each model was adjusted for these three factors. To examine potentially differential associations across study groups, interaction terms were applied between group status and PSP, CBCL, and CGAS respectively. We used a significance level of 5%, and effect sizes were calculated using Cohen’s *d* (between-group comparisons) and partial eta squared (*R*^2^) (associations). All analyses were performed with Stata 17 statistical software (StataCorp, 2021).

## Results

### Sample characteristics

In the VIA 11 Study, 160 primary caregivers from families with schizophrenia, 95 primary caregivers from families with bipolar disorder, and 177 primary caregivers from the population-based control families filled in the FAD-GFS. One primary caregiver from the control group had missing items (1 missing item, i.e., < 9% missing items), whereas no primary caregivers from the groups with parental schizophrenia or bipolar disorders had missing items on the FAD-GFS. Of these families, we obtained data on CGAS from 157 children at FHR-SZ, 95 at FHR-BP, and 174 controls and data on CBCL from 157 children at FHR-SZ, 95 at FHR-BP, and 172 controls. The primary caregivers in the FHR-SZ and the FHR-BP groups displayed significantly lower levels of social functioning (PSP), a significantly lower prevalence of employment, and were more likely to be a single caregiver compared with the primary caregivers in the control group (all *p* values < .001) but did not differ from each other (Table 1). Children at FHR-SZ (*p* < .001) and FHR-BP (*p* < .01) demonstrated lower levels of global functioning (CGAS) and more total problem behavior (CBCL) including both more internalizing and more externalizing problem behavior compared with controls (*p* values < .01) but did not differ from each other on either dimension (Table 1).

**Table 1.** Demographic and clinical characteristics of 432 primary caregivers and their pre-adolescent offspring in families with parental schizophrenia or bipolar disorder and population-based controls

					Pairwise comparisons		
Primary caregiver	PBC *n* = 177	BP*n* = 95	SZ*n* = 160	p	BP vs. PBC*p*	SZ vs. PBC*p*	SZ vs. BP*p*
Assigned female at birth *n* (%)	154 (87.01)	78 (82.11)	137 (85.63)	.549			
Age at child’s birth Mean (SD)	32.13 (4.12)	31.80 (5.87)	30.69 (6.70)	.053			
Primary caregiver is an index *n* (%)	112 (63.28)	51 (53.68)	71 (44.38)	**.002**	.124	**.001**	.150
Single caregiver *n* (%)	25 (14.29)	39 (41.05)	49 (30.63)	**< .001**	**< .001**	**< .001**	.090
PSP Mean (SD)	83.30 (10.19)	71.83 (15.46)	70.90 (16.31)	**< .001**	**< .001**	**< .001**	.864
Employed or studying *n* (%)	171 (97.16)	72 (75.79)	121 (76.10)	**< .001**	**< .001**	**< .001**	.955
Education							
Primary/lower secondary, *n* (%)	25 (14.29)	16 (17.02)	42 (26.25)	**.030**	.577	.14	.065
Upper secondary, vocational, short-cycle tertiary, *n* (%)	53 (30.11)	23 (24.47)	48 (30.00)				
Bachelor degree, equivalent or higher, *n* (%)	98 (56.00)	55 (58.51)	70 (43.75)				
**Child**							
Assigned female at birth *n* (%)	82 (46.33)	43 (45.26)	78 (48.75)	.842			
Age Mean (SD)	11.94 (0.22)	11.94 (0.22)	11.97 (0.23)	.261			
CGAS, Mean (SD) (PBC: *n* = 174; FHR-BP: *n* = 95; FHR-SZ: *n* = 157)	75.11 (13.99)	68.98 (14.67)	64.82 (15.56)	**< .001**	**.003**	**< .001**	.077
CBCL Total Score, Mean (SD) (PBC: *n* = 172; FHR-BP: *n* = 95; FHR-SZ: *n* = 157)	12.78 (12.69)	20.89 (20.45)	23.84 (20.60)	**< .001**	**.001**	**< .001**	.408
CBCL Internalizing Score, Mean (SD) (PBC: *n* = 172; FHR-BP: *n* = 95; FHR-SZ: *n* = 157)	4.70 (5.01)	7.05 (6.85)	6.99 (6.43)	**< .001**	**.007**	**.002**	.996
CBCL Externalizing Score, Mean (SD) (PBC: *n* = 172; FHR-BP: *n* = 95; FHR-SZ: *n* = 157)	2.43 (3.19)	4.61 (5.78)	5.95 (6.62)	**< .001**	**.004**	**< .001**	.125

*Notes:* Bold face values indicate *p* values < .05. PBC, population-based controls; BP, bipolar disorder; SZ, schizophrenia; PSP, Personal and Social Functioning Scale; CGAS, Children’s Global Assessment Scale; FHR, familial high-risk; CBCL, Child Behavior Checklist School-Age Version.

*Drop-out analyses.* In the families of the VIA 11 study, who were not assessed with the FAD-GFS (FHR-SZ: *N* = 12; FHR-BP: *N* = 6; controls: *N* = 3), the primary caregivers had significantly lower levels of social functioning (PSP, *p* < .001) and the children had significantly lower levels of global functioning (CGAS, *p* = .002) as well as higher levels of psychopathology (CBCL Total, *p* = .01) compared with the primary caregivers and children in the families who participated.

### Family functioning

**Figure 1.**
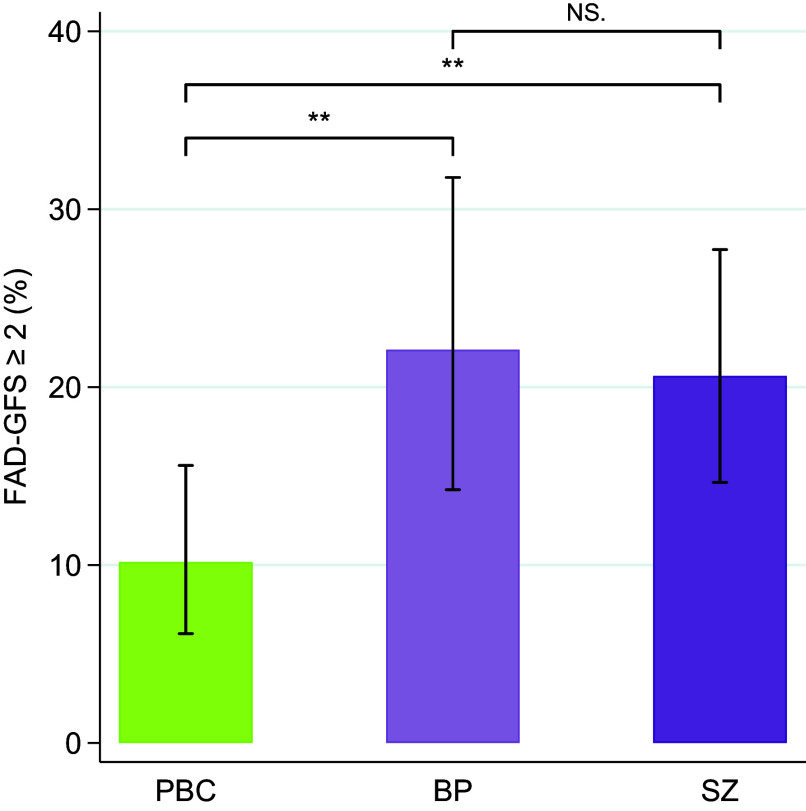
Prevalence of families with unhealthy family functioning (FAD-GFS scores ≥2). *Note:* **p values < .01. Error bars indicate 95% CI. NS, non-significant; PBC, population-based controls; BP, bipolar disorder; SZ, schizophrenia; FAD-GFS, Family Assessment Device – General Functional Scale.

The primary caregivers in the families with parental schizophrenia (Cohen’s *d* = 0.29, *p* = .002) and with parental bipolar disorder (Cohen’s *d* = 0.34; *p* = .004) reported significantly poorer family functioning (FAD-GFS) compared with the primary caregivers in the control group but did not differ from each other (Cohen’s *d* = 0.03; *p* = .783) (Table 2). A significantly higher prevalence of the primary caregivers in the families with parental schizophrenia (Cohen’s *d* = 0.29; *p* = .007) and parental bipolar disorder (Cohen’s *d* = 0.34; *p* = .007) also reported a level of family dysfunction that was above clinical cut-off compared with the prevalence in the control group, but they were non-significantly different from each other (Cohen’s *d* = 0.04; *p* = .780) (Table 2; Figure 1). Covarying for child age, child sex, and primary caregiver’s level of education did not change any of the significant between-group effects (data not shown).

**TABLE 2.** Family functioning was reported by 432 primary caregivers in families with parental schizophrenia or bipolar disorder and population-based controls

					Pairwise comparisons		
	PBC *n* = 177	BP *n* = 95	SZ *n* = 160	p	BP vs. PBC *p* Cohen’s *d*	SZ vs. PBC *p* Cohen’s *d*	SZ vs. BP *p* Cohen’s *d*
FAD-GFS Mean (SD)	1.45 (0.39)	1.60 (0.47)	1.59 (0.41)	**.002**	**.004** 0.34	**.002** 0.29	.783 0.03
FAD-GFS above clinical cut-off (≥2), *n* (%)	18 (10.27)	21 (22.11)	33 (20.62)	**.010**	**.007** 0.34	**.007** 0.29	.780 0.04

*Note:* Bold face values indicate *p* values < .05. PBC, population-based controls; BP, bipolar disorder; SZ, schizophrenia; FAD-GFS, Family Assessment Device – General Functional Scale; higher scores reflect poorer functioning. Above clinical cut-off scores (≥2) indicate unhealthy family functioning.

### Associations between family functioning and levels of child global functioning, child psychopathology, and the primary caregiver’s social functioning

**Figure 2.**
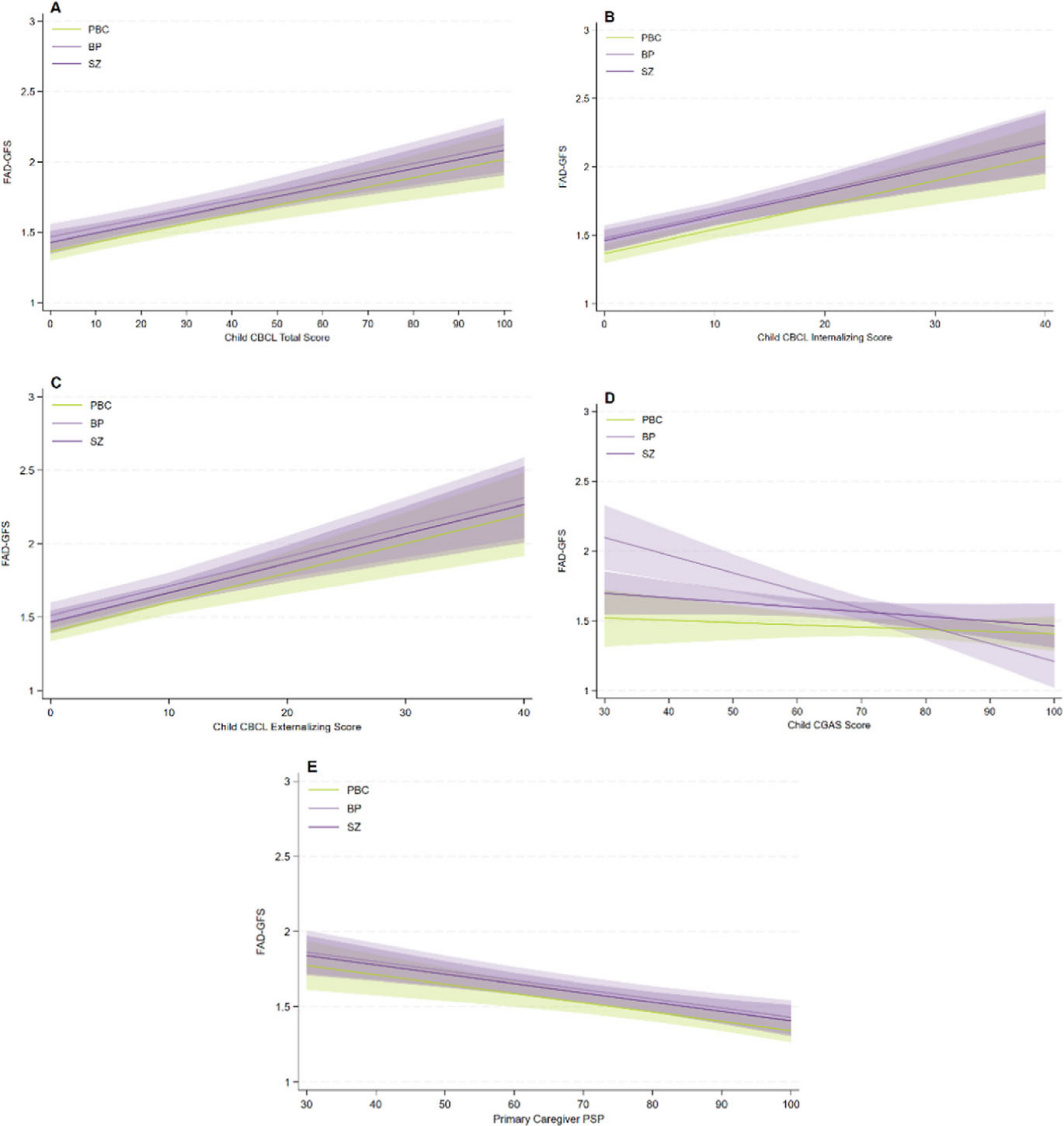
(a–e) Associations between family functioning and levels of child global functioning, child psychopathology, and the primary caregiver’s social functioning. *Note*: All results are adjusted for familial high-risk status. Error bars indicate 95% CI. FAD-GFS, Family Assessment Device – General Functional Scale (higher scores reflect poorer functioning); CBCL, Child Behavior Checklist School-Age Version (higher scores indicate higher levels of psychopathology); CGAS, Children’s Global Assessment Scale (higher scores denote better functioning); PSP, Personal and Social Functioning Scale (higher scores denote better functioning); PBC, population-based controls; BP, bipolar disorder; SZ, Schizophrenia.

Higher levels of child psychopathology (CBCL total score) were significantly associated with poorer family functioning (FAD-GFS) (β = 0.0065, [95% CI: 0.004;0.009], *t* = 5.89, *p* < .001, *R*^2^ = 0.076) in a model adjusted for high-risk status (Figure 2a). Adding familial high-risk status as interaction term did not significantly improve the fit of the model, rendering the association non-significantly different across the three study groups (*p* = .08). The same pattern applied for child internalizing psychopathology (the CBCL internalizing score), where more internalizing psychopathology was significantly associated with poorer family functioning (FAD-GFS) (β = 0.0178, [95% CI: 0.011;0.024], *t* = 5.37, *p* < .001, *R*^2^ = 0.064) in a model adjusted for high-risk status, as well as for child externalizing psychopathology (the CBCL externalizing score), where more externalizing psychopathology was significantly associated with poorer family functioning (FAD-GFS) (*β* = 0.0200, [95% CI: 0.013;0.027], *t* = 5.31, *p* < .001, *R*^2^ = 0.063) in a model adjusted for high-risk status (Figures 2b,c). Adding familial high-risk status as an interaction term did not significantly improve the fit of the model for either the CBCL internalizing score (*p* = .10) or the CBCL externalizing score (*p* = .11), rendering the associations non-significantly different across the three study groups. There was a significant interaction between global functioning of the child (CGAS) and familial high-risk status (*p* = .006, *R*^2^ = 0.041). This association was significantly different in the FHR-BP compared with the control group (*β* = −0.0127, [95% CI: −0.018;-0.007], *t* = −4.45, *p* < .001) but not in the FHR-SZ compared with control group (*β* = −0.0034, [95% CI: −0.0074;0.001], *t* = −1.61, *p* = .11) (Figure 2d). In families with parental bipolar disorder, lower global functioning of the child was associated with poorer family functioning. Finally, we found a significant association between poorer social functioning (PSP) of the primary caregiver and poorer family functioning (FAD-GFS) (*β* = −0.0062, [95% CI: −0.009;-0.003], *t* = −4.35, *p* < .001, *R*^2^ = 0.042) in a model adjusted for high-risk status (Figure 2e). This association was not significantly different across the three study groups (*p* = .74). When adjusting for child age, child sex, and primary caregiver’s level of education in each model, there was no significant effect of these three factors (data not shown).

## Discussion

In this prospective and nationwide familial high-risk cohort study of families with 11-year-old children, we found poorer family functioning and a higher prevalence of clinically significant family dysfunction reported by the primary caregiver in families with parental schizophrenia or bipolar disorder compared with population-based controls. The two familial high-risk groups did not differ from each other. Across study groups, poorer family functioning was associated with higher levels of child psychopathology, i.e., both internalizing, externalizing, and total psychopathology, and with lower levels of social functioning of the primary caregiver, whereas it was differentially associated with child global functioning, where it was only associated with poorer levels of child global functioning in the families with parental bipolar disorder.

The findings of the current study corroborate previous findings of increased risk of poorer family functioning in families with severe parental mental disorders (Friedmann et al., 1997; Sell, Daubmann, et al., 2021; Shalev et al., 2019). However, the prevalence of families in the current study, where the primary caregiver reported clinically significant family dysfunction, was considerably lower (FHR-BP = 22%; FHR-SZ = 21%) compared with the prevalence (57%) in a Swedish study of families with either parental depression, anxiety, or bipolar disorder using the same measure (Nordh et al., 2022). This difference may be due to a higher prevalence of remission among the parents with schizophrenia or bipolar disorder in our cohort, who were recruited through the Danish registers, whereas the Swedish sample was recruited from psychiatric clinics. Another explanation for this difference in prevalence could be that the vast majority of parents reporting family functioning in the Swedish study were patients (92.1%), whereas only around half of the primary caregivers in the current study were index parents (schizophrenia = 44.4%; bipolar disorder = 53.7%). Finally, a 20-year follow-up study of parents with a first episode of schizophrenia spectrum disorder showed prevalence rates of family dysfunction (28.6%) that were closer to that of the families with parental schizophrenia in the current study (Hansen et al., 2024).

Our findings that poorer family functioning is associated with higher levels of child psychopathology in both high- and low-risk children are consistent with previous findings from a representative, cross-sectional cohort study in the general population (Wille et al., 2008) and several cross-sectional (Daches et al., 2018; Sell, Barkmann, et al., 2021; Wiegand-Grefe et al., 2019) and longitudinal (Shalev et al., 2019) studies of families with parental severe mental disorders except from one cross-sectional study of families with parental depression, bipolar, or anxiety disorders, where family functioning did not correlate with child psychopathology (Nordh et al., 2022). Further, previous evidence suggests that poorer family functioning is associated with lower levels of overall adjustment in adult individuals with depression (Staccini et al., 2015), which supports our finding that poorer social functioning of the primary caregiver was associated with poorer family function across all groups. Further, we also found that poorer child global functioning was associated with poorer family functioning in the group of children at FHR-BP. The latter association was not found in children at FHR-SZ or in the control group. Although speculative, this may be explained by a higher prevalence of children at FHR-BP living in a single-caregiver household (although not significantly higher than children at FHR-SZ) with potentially fewer parental resources, which in a previous study of the current cohort has been found to affect the home environment and the ability to sufficiently stimulate the child (Thorup et al., 2022).

Due to the cross-sectional evidence of the current study, the direction of effects in the investigated associations is potentially bidirectional, which must be considered in the interpretation of the results. For example, child psychopathology may affect family functioning and vice versa (Berg-Nielsen et al., 2002). In terms of generalizability of the current study results, we have previously investigated the representativity of our cohort (Krantz et al., 2022) and found poorer parental and child functioning and a higher prevalence of risk factors in non-participants compared with participants retrieved from the original data extracted from the Danish registers (Supplementary Figure S1). Therefore, the identified differences in family functioning between families with parental schizophrenia or bipolar disorder and controls may potentially be even more pronounced.

### Potential clinical implications

Due to the combination of increased genetic risk of mental disorders and a potential environmental risk factor such as family dysfunction, future studies should further investigate brief, low-cost, and low-risk screening instruments for family dysfunction such as the FAD-GFS in families with parental schizophrenia or bipolar disorder. Future studies should also investigate interventions targeting the familial high-risk sub-groups with a clinically significant level of family dysfunction. Family-focused therapy, psychoeducational multi-family group treatment, and family group cognitive-behavioral preventive interventions have proven effective in youth and young adults at clinical high risk for psychosis (O’Brien et al., 2007, 2014), symptomatic youth at familial high risk of bipolar disorder (Miklowitz et al., 2013, 2020), and children at familial high risk of major depressive or dysthymic disorder (Compas et al., 2009; 2015) and could therefore be viable remedies to apply in future intervention studies of preadolescent children in families with parental schizophrenia or bipolar disorder. Also, whole-family intervention approaches have shown promising effects on child mental health and family outcomes in families with parental mental illness, but more high-quality research is needed (Moltrecht, Lange, Merrick, & Radley, 2024). Finally, impaired parental functioning in key areas such as social responsiveness and neurocognitive functions have previously been documented in parents with schizophrenia and bipolar disorder in the current cohort (Greve et al., 2022; Veddum et al., 2023) and may also be relevant targets for intervention studies that aim to investigate methods to improve family functioning. Due to potential bidirectionality in the relationship between family functioning and child psychopathology and global functioning, future intervention studies must enable the differentiation of various effects. In terms of research perspectives, an ongoing follow-up study of the current cohort will provide longitudinal evidence on the development of family functioning reported by primary caregivers and their 15-year-old children. This will also allow for the investigation of potential mediators as well as the predictive value of baseline family functioning in relation to longitudinal outcomes.

### Strengths and limitations

The strengths of the current study include the register-based and nationwide recruitment of families with parental schizophrenia or bipolar disorder and the matched population-based control group. We used a low-cost, low-effort, low-risk, and well validated questionnaire that could easily be applied in clinical practice. Finally, the current study fills in a gap in the existing literature on family functioning in families with parental schizophrenia or bipolar disorder, which is limited and includes small sample sizes, a broad spectrum of mental disorders, and a lack of a control group (Sell, Barkmann, et al., 2021; Sell, Daubmann, et al., 2021). This study also has several limitations. Due to the cross-sectional nature of our analyses and potential bidirectional effects, inferences regarding causality cannot be drawn. Second, due to the FAD being designed for family members ≥ the age of 12 (Miller et al., 2000), we only used primary caregiver-reported measures of family functioning and did not capture the children’s perception hereof. Previous evidence suggests that children may report less family dysfunction than parents with a mental disorder but comparable to the ratings of partners to the index parents (Sell, Daubmann, et al., 2021). Third, our drop-out analyses revealed poorer parental social functioning, poorer child global functioning, and more child psychopathology in the non-participating families compared with the families who were assessed with the FAD-GFS. This bias may indicate that our main findings would have been more pronounced if all families in the VIA 11 study had participated in the FAD-GFS. Fourth, only families with parents born in Denmark were included, which limits the extension of results to children with a more diverse cultural background. Fifth, the proportion of the shared variance in the investigated associations was relatively small (ranging between 4.4% and 7.6%), which must be taken into consideration when interpreting the results.

## Conclusions

Primary caregivers in families with parental schizophrenia or bipolar disorder reported poorer family functioning and a higher prevalence of a clinically significant level of family dysfunction compared with population-based controls. Poorer family functioning was associated with higher levels of child psychopathology and poorer parental social functioning irrespective of group status but only with a lower level of child global functioning in families with parental bipolar disorder. The potentially detrimental effects of the interaction of both genetic and environmental risk such as family dysfunction in these children at familial high risk of schizophrenia or bipolar disorder warrant early detection of families with family dysfunction above clinical cutoff, and the current study may inform future intervention studies and programs.

## Supporting information

Hemager et al. supplementary material Hemager et al. supplementary material

## Supplementary material

The supplementary material for this article can be found at http://doi.org/10.1017/S0033291725000200.

## Code availability statement

According to GDPR rules, data can be accessed by researchers upon data agreement application and approval.
