# Supplementary material for: Family functioning in families with 11-year-old children at familial high risk of schizophrenia or bipolar disorder and population-based controls: The Danish High Risk and Resilience Study VIA 11
Source: Psychol Med. 2025 Feb 25;55:e62. doi: 10.1017/S0033291725000200 (PMC12080666; doi:10.1017/S0033291725000200)
Supplement: Hemager et al. supplementary material [file S0033291725000200sup001.docx]

Data extracts from Danish National Registers^a^ of children with FHR-SZ^b,c^, FHR-BP^d^ and up to 10 Controls^e^ for each case, born between 1 September 2004 and 31 August 2009 **(N = 24,706)**

Children who were too old or too young

**(N = 5,376)**

Children retrieved as matched controls to FHR-BP

**(N = 7,373)**

N=5 376

Children eligible for inclusion in VIA 7

**(N = 11,957)**

Controls

**(N = 10,110)**

FHR-BP

**(N = 774)**

FHR-SZ

**(N = 1,073)**

No contact attempted (N = **560)**

No contact attempted **(N = 9,791)**

No contact attemted **(N = 663)**

Attempted contact

**(N = 410)**

Attempted contact

**(N = 319)**

Attempted contact

**(N = 214)**

Non-respondents **(N = 92)** Declined **(N = 116)**

Non-respondents **(N = 40)** Declined **(N = 79)**

Non-respondents **(N = 40)** Declined **(N = 54)**

FHR-SZ included in the VIA 7 study

**(N = 202)**

FHR-BP included in the VIA 7 study

**(N = 120)**

Controls included in the VIA 7 study

**(N = 200)**

Declined **(N = 19)**

Declined **(N = 15)**

Declined **(N =23)**

FHR-SZ included in the VIA 11 study (**N=179**)

Controls included in the VIA 11 study (**N=181**)

FHR-BP included in the VIA 11 study (**N=105)**

Excluded^f^ **(N = 10)**

Excluded^f^ **(N = 4)**

Excluded^f^ **(N =19)**

FHR-SZ included in the present study of family functioning

(**N=160**)

FHR-BP included in the present study of family functioning

(**N=95)**

)

FHR-SZ included in the present

study of family functioning

(**N=177**)

**^a^ Danish National Registers:** Danish Civil Registration System and Danish Psychiatric Central Research Register.

**^b^ FHR-SZ:** Children at familial high risk of schizophrenia spectrum disorders.

**^c^ Double diagnosed parents:** Parents with diagnoses of schizophrenia and bipolar disorder were assigned to the schizophrenia high risk group as per the ICD-10 hierarchy.

**^d^ FHR-BP:** Children at familial high risk of bipolar disorder.

**^e^ Controls:** Population-based control children of parents with no diagnoses of schizophrenia spectrum disorders or bipolar disorder.

^f^Reasons for exclusion are participation of sibling, recent pariticpiation in another study of FAD, and partial participation due to e.g. limited time resources
